# An amphibian chemical defense phenotype is inducible across life history stages

**DOI:** 10.1038/s41598-017-08154-z

**Published:** 2017-08-15

**Authors:** Gary M. Bucciarelli, H. Bradley Shaffer, David B. Green, Lee B. Kats

**Affiliations:** 1 UCLA Department of Ecology and Evolutionary Biology, University of California, Los Angeles, USA; 2 La Kretz Center for California Conservation Science, Charles E. Young Drive, Los Angeles, CA 90095 USA; 3 Pepperdine University, Natural Science Division, Pacific Coast Highway Malibu, Los Angeles, CA USA

## Abstract

Inducible phenotypic responses to environmental variation are ubiquitous across the tree of life, but it remains an open question whether amphibian chemical defense phenotypes are inducible. Tetrodotoxin (TTX) is a key chemical defense trait in North American and Eurasian newts (Salamandridae). We tested if TTX can be induced by exposing populations of adult and larval California newts (*Taricha torosa*) to sustained stressful conditions while longitudinally quantifying TTX concentrations. Adult newts rapidly increased chemical defenses in response to simulated predator attacks and consistently maintained elevated TTX concentrations relative to wild, non-captive individuals. We also found that laboratory-reared larvae maintained chemical defenses nearly three-fold greater than those of siblings reared in streams. Collectively, our results indicate that amphibian chemical defenses are not fixed. Instead, toxins are maintained at a baseline concentration that can quickly be increased in response to perceived risk with substantial increases to toxicity. Therefore, it is crucial that inducible variation be accounted for when considering ecological dynamics of chemically defended animals and coevolutionary predator-prey and mimic-model relationships.

## Introduction

Phenotypic variation, or plasticity, is a ubiquitous strategy taxa use to cope with variable environmental conditions and ecological challenges^1–4^ with demonstrated adaptive significance^5–7^. Some of the most remarkable plastic phenotypes are defensive traits such as spines, armors, and toxins that species can rapidly deploy in response to predator or herbivore attacks, physical harm, or increased stress^8–11^, thereby reducing predation vulnerability and mortality. Inducible defenses typically have a baseline, or constitutive level of expression^12^ that can rapidly increase when triggered via chemical, visual, or tactile stimuli. While most widely explored as a feature of plant defenses, the potential for amphibians to amplify chemical defense phenotypes remains entirely overlooked even though poison glands are a defining characteristic of the group.

Populations of wild-caught North American newts (family Salamandridae, genera *Taricha* and *Notophthalmus*) possess the neurotoxin tetrodotoxin (TTX), which they maintain under captive conditions. Broad geographic descriptions of salamandrid chemical defense concentrations show wide-ranging variation among populations^13–16^. However, the mechanisms underlying this variation remain unknown, including the fundamental question of whether newts produce TTX via microbial symbionts or if the genus is uniquely capable among metazoans of de novo synthesis. Such ambiguities present a challenge to understanding both the ecological and evolutionary dynamics of amphibian chemical defenses.

Current theories maintain that amphibian chemical defenses are a fixed evolutionary response to local predation pressure, incapable of short-term environmental responses, and stable within wild individuals and populations over ecological time scales^15, 17, 18^. In fact, the textbook example of an evolutionary “arms race” between toxic amphibian prey and resistant predators is based in part on the assumption that salamandrid chemical defenses are not inducible. While reasonable, there is little empirical evidence to support this model. Previous studies indirectly hint at the possibility that TTX may be inducible^19, 20^, and a recent study indicated that population average TTX concentrations are capable of fluctuating by orders of magnitude and within the same wild individuals over days or seasons^21^.

In this study, we experimentally evaluated the responsiveness of adult and larval amphibian chemical defenses in a species of newt (California newt, *Taricha torosa*). *Taricha* is the most toxic genus of salamanders currently known and has served as a model system to study the evolution of tetrodotoxin. To test the lability of chemical defenses, we simulated predator attacks^22–24^ by experimentally sampling toxic male *T. torosa* from two geographically disparate populations subjected to *ex situ* and *in situ* captive conditions. Isolated and quantitated TTX from these individual skin samples was compared to those of simultaneously sampled wild counterparts from their respective populations. To determine if larval chemical defenses are affected by environmental variation, we bifurcated egg masses and reared siblings from corresponding halves under stream or laboratory conditions, longitudinally collected larvae, and evaluated TTX concentrations of siblings through time. Results indicate that adult chemical defenses are highly inducible and that larvae differentially maintain or invest in TTX depending on the environment. Collectively, chemical defenses in *T. torosa*, and very likely many other amphibians can be induced from constitutive, or baseline levels to much higher concentrations. As such, models explaining the evolution and ecology of amphibian chemical defenses, coevolutionary relationships, and mimic-model systems that assume chemical defenses cannot be induced, must account for this previously unrecognized source of variation.

## Results

**Figure 1:**
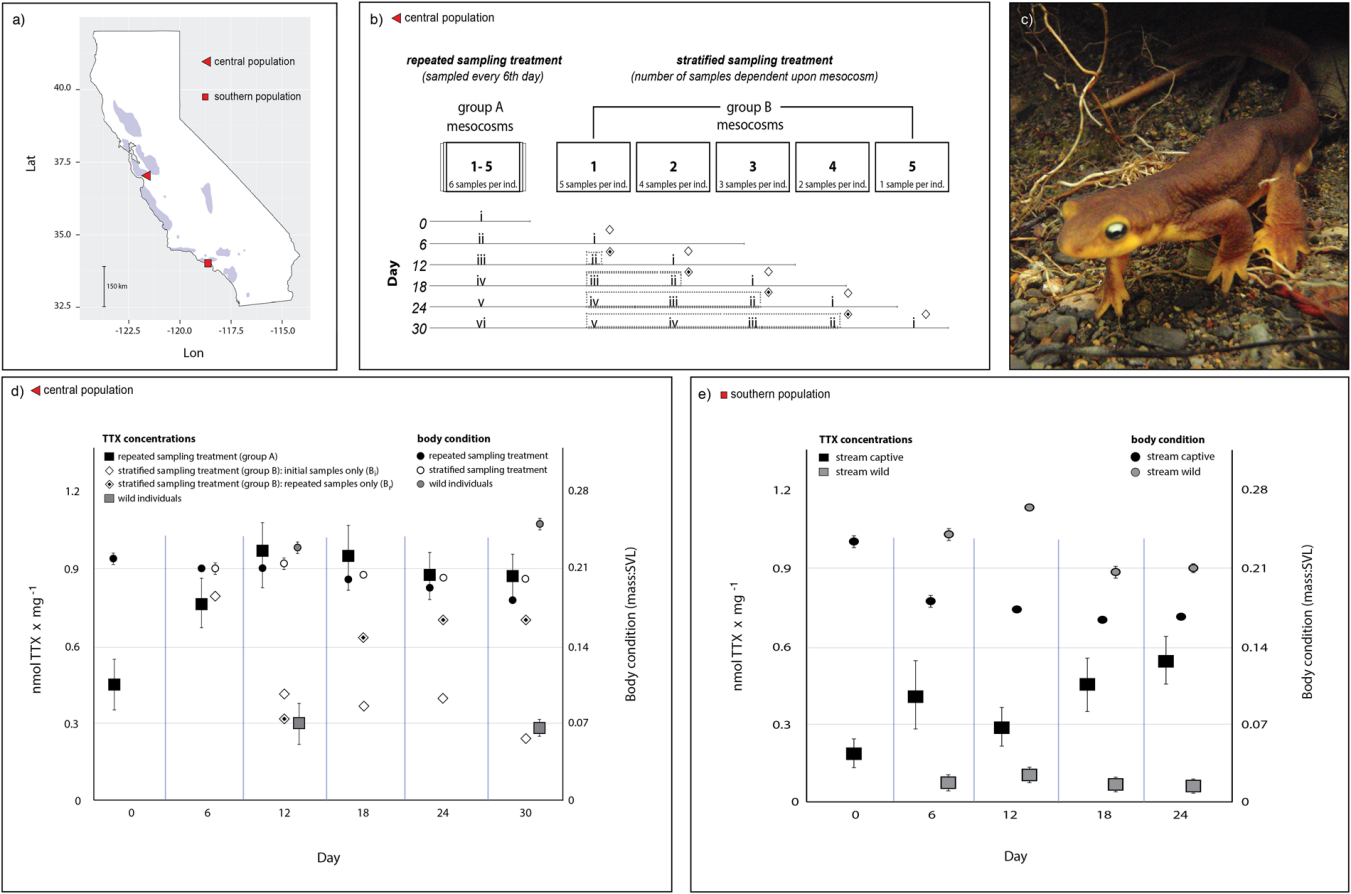
Changes in chemical defense concentrations of toxic newt populations in California. **(a**) A map of California created in *R* ^61^ using *ggplot2* (v.2.20)^64^ shows the current distribution of the focal species, *Taricha torosa* and marked locations of the study populations. (**b**) A schematic of the experimental sampling design for the *ex situ* experiment with newts from central California. Time is represented from day 0–30 and is associated with a horizontal line to show which individuals in a group were sampled on that day. Roman numerals represent the sample number and indicate whether the sample is initial (i) or a repeat (ii–vi) for newts in the stratified sampling experiment (group B). Diamonds represent initial samples (hollow) or repeated samples (dotted) and correspond to data in panel d. Dotted lines encompass the mean values depicted in panel d. (**c**) An underwater image of a breeding male California newt (photo by G.M. Bucciarelli). (**d**) The mean body condition and TTX concentrations (nanomoles mg^−1^ tissue, ±s.e.m.) of experimentally sampled and wild newts from the central California population during the *ex situ* experiment show that chemical defenses increased rapidly as a result of repeated sampling. Mean chemical defenses of the repeatedly sampled newts in group A (*P* = 0.01) and repeatedly sampled newts in group B (B_r_ - mean values encircled within dashed boxes in panel b and marked with dotted diamonds in panel b) increased significantly through time (*P* = 0.02) whereas mean chemical defenses of initially-sampled individuals from the stratified sampling treatment (B_i_, mean values; marked in panel b with hollow diamonds) did not significantly increase (*P* = 0.10) or differ from wild, non-captive individuals (*P* = 0.12). Mean body condition values (±s.e.m.) of captive and non-captive newts suggest potential costs associated with increased chemical defenses, but the predictor was not significant (*P* = 0.91). (**e**) Adult male mean TTX concentrations (±s.e.m.) of *in situ* newts from the southern California population significantly increased through time (*P* = 0.004) and chemical defenses significantly differed relative to cohabitating wild, non-captive newts sampled on the same day (*P* = 0.01). Although notably different, body condition (±s.e.m.) was not a significant predictor (*P* = 0.098). Vertical blue bars in both panels d and e separate data of each sampling day.

**Figure 2:**
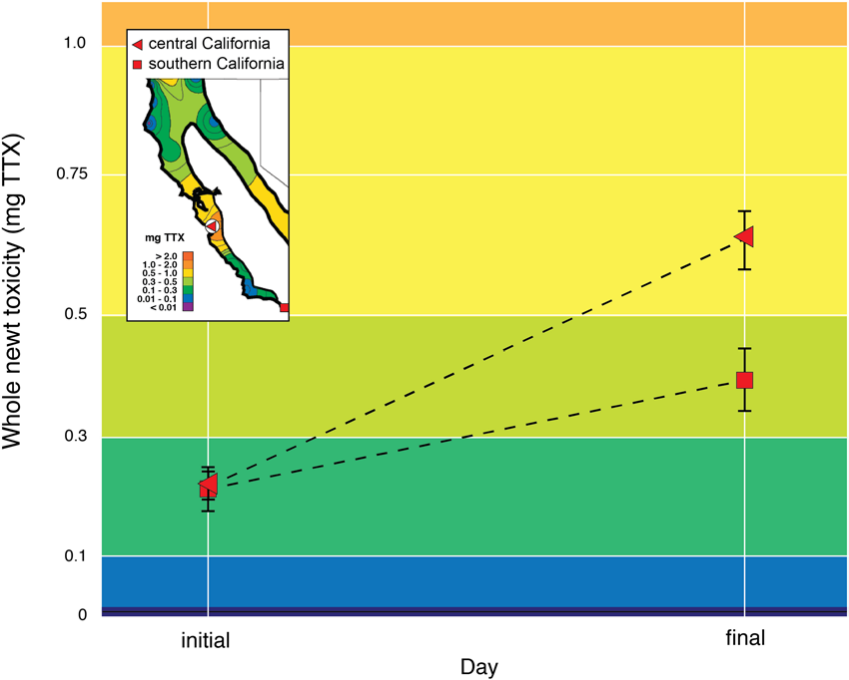
Induced toxicity of male chemical defenses. Changes in estimated toxicity (mg TTX) of repeatedly-sampled California newts show that initial toxicity significantly increased in central California (repeated sampling treatment/group A: paired *t* test; n = 15; *P* = 0.01) and stream-captive southern California males (paired *t* test; n = 9; *P* = 0.02). Error bars represent s.e.m. after accounting for between-subject variability^58^. The inset map (appropriated from^18^ and modified to include point localities of our study populations) shows previously described estimates of newt toxicity across the distributional range of *Taricha* in California^18^. The color index shows toxicity from low (blues) to high (orange). Induced changes measured in our experimental populations span much of the toxicity range in wild populations.

We found that the central California population rapidly increased and maintained elevated TTX concentrations in response to simulated predator attacks (Fig. 1d, black squares), indicating that chemical defenses can be modulated over a relatively short temporal scale. Observed increases in TTX concentrations across experimental groups (repeated and stratified treatments, Fig. 1b,d) could not be explained by aquatic conditions (water temperature: β = 0.01, SE = 0.009, *P* = 0.23; pH: β = −0.31, SE = 0.20, *P* = 0.13) or by differences in body condition (β = −2.01, SE = 1.77, *P* = 0.25). However, changes were explained by experimental group and temporal dynamics (Fig. 1d; *group (reference group A*): β = −0.27, SE = 0.12, *P* = 0.034; *time*: β = 0.01, SE = 0.005, *P* = 0.045). Independent analyses by group showed that the chemical defenses of newts in the repeated sampling treatment (group A) differed over the duration of the study (*time*: β = 0.007, SE = 0.003, *P* = 0.016), while those in the stratified sampling treatment (group B) did not (*time*: β = 0.007, SE = 0.005, *P* = 0.17). Moreover, when we evaluated whether chemical defenses of initially sampled (B_i_) and repeatedly sampled (B_r_) males in the stratified sampling treatment (group B) (Fig. 1d; compare hollow and dotted diamonds) differed, we found that TTX concentrations temporally varied among repeatedly-sampled males (B_r_: β = 0.014, SE = 0.006, *P* = 0.025), but not initially-sampled males (B_i_ β = −0.023, SE = 0.013, *P* = 0.103). Thus, individuals that remained in captivity without being sampled (open diamonds, Fig. 1d), regardless of the duration, did not change their chemical defenses, whereas those that were repeatedly sampled in this same group did. This result indicates that repeated sampling, not the response to captivity modulated TTX. Finally, when we compared chemical defenses of the initial samples from captive individuals in the stratified sampling group (B_i_) to those of wild, non-captive males located in their native habitat (Fig. 1d, open diamonds compared to grey squares) we found no significant differences between TTX concentrations (reference group B_i_: β = −0.18, SE = 0.11, *P* = 0.12) or body condition (β = 0.21, SE = 2.12, *P* = 0.91) of these two groups, suggesting that although chemical defenses in captive newts may have initially spiked (likely a result of marking individuals), captive and wild newts maintained similar baseline levels of TTX. An assessment of the initial and final chemical defense concentrations (nmol TTX mg^−1^) and estimated newt toxicity (total dermal mg TTX) of newts in the repeated sampling treatment (group A) showed that chemical defenses and toxicity significantly increased (*chemical defenses*: 0.49 (±0.09) − 0.87 (±0.16) nmol TTX (±s.e.m.), two-tailed paired *t* test: n = 15, *t* = −4.63, *df* = 14, *P* < 0.001; *newt toxicity*: 0.24 (±0.05) − 0.62 (±0.09) mg TTX (±s.e.m.), *t* = −2.63, *P* = 0.01; Fig. 2).

As with the central California population, chemical defenses of repeatedly-sampled newts from the southern California population increased through time (β = 0.03, SE = 0.01 *P* = 0.004, Fig. 1e) and significantly differed from wild males co-occurring and simultaneously sampled in the same breeding stream (*group*: β = −1.31, SE = 0.51, *P* = 0.01). Although variable, body condition could not explain changes in TTX concentrations (β = −5.71, SE = 5.44, *P* = 0.30). Individual chemical defenses on average tripled (Fig. 1e) and estimates of toxicity doubled (Fig. 2b). Both significantly increased over the duration of the study (*chemical defenses*: 0.32 (±0.13) − 0.50 (±0.13) nmol TTX (±s.e.m.), two-tailed paired *t* test: *n* = 9, *t* = −2.40, *df* = 8, *P* = 0.04; *newt toxicity*: 0.22 (±0.07) − 0.39 (±0.11) mg TTX (±s.e.m.), *t* = −2.73, *P* = 0.02; Fig. 2). Overall, each Monte Carlo based permutation test fully supported the results of each model for each population.

**Figure 3:**
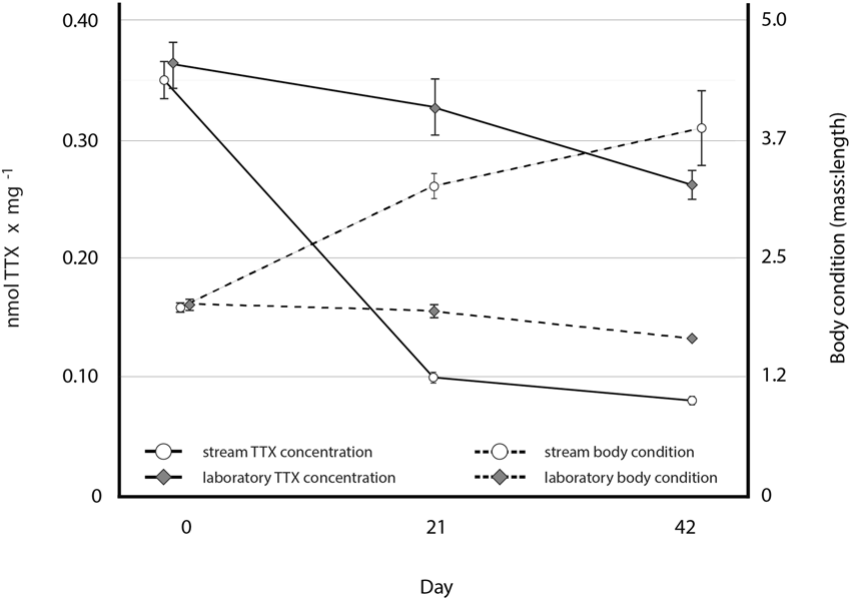
The TTX concentrations and body condition of larval siblings reared in two environments across time. At hatch (day 0), siblings showed nearly identical mean TTX concentrations and body condition. However, sibling responses differed through time between environments. In general, laboratory-reared larvae maintained greater TTX concentrations, but had lower body condition, whereas stream-reared siblings responded oppositely. The three-way interaction term between location (stream or laboratory), time, and body condition was significant (*P* = 0.005), suggesting that there are trade-offs between larval body condition and chemical defenses.

When we reared larval siblings in different environments, we found that TTX concentrations and larvae size significantly differed between laboratory and stream groups through time (location*body condition*days, β = 0.65, SE = 0.23, *P* = 0.005; post-hoc Chi square goodness of fit test *P* = 0.004; permutation test *P* = 0.006). Laboratory-reared larvae had higher TTX concentrations and lower body condition, whereas stream-reared larvae showed the opposite pattern (Fig. 3), suggesting that larvae differentially invested in chemical defenses due to the environment. When we tested whether developmental stages differed at each sampling point between groups, we detected significant differences between groups at the intermediate and final sampling periods (*day* 21: *t* = 8.14, *df* = 54, *P* < 0.001; *day* 42: *t* = 2.84, *df* = 47, *P* = 0.006). However, comparisons of larvae development on days 21 (stream-reared) to 42 (laboratory-reared) did not differ (*t* = 0.81, *df* = 55, *P* = 0.42), but their TTX concentrations did (*t* = 6.95, *df* = 74, *P* < 0.001; *stream* $\bar{x}$ = 0.10 nmol/mg; *laboratory* $\bar{x}$ = 0.26 nmol/mg). Therefore, divergent chemical defenses between siblings reared in the two environments is not likely the result of developmental differences.

## Discussion

The rapid increase of TTX concentrations and elevated toxicity we induced in adult California newts reveal that the trait is plastic, implying that chemical defenses in wild populations are not shaped solely by evolutionary processes^13, 18^, but instead react to ecological dynamics and environmental conditions. There is little understanding of how environmental variability may affect animal chemical defense traits, even though these traits tend to heavily mediate ecological and evolutionary trajectories^25^. The ability to rapidly activate chemical defenses in response to environmental stimuli is well documented in at least four of the primary biological kingdoms. Phytoplankton possess a precursor enzyme dimethylsulfoniopropionate lyase that is rapidly upregulated in response to physiological stressors and herbivory to produce dimethyl sulfide (DMS) and acrylate, both of which serve as chemical defenses against grazers^26^. Rapid inductions of chemical defenses also occur in bacteria^27^ and are well-characterized across a diversity of plant taxa^28^. We found amphibians can induce chemical defenses in similar and predictable ways. Like many other taxa^9, 29, 30^ this response appears to be elicited by perceived risk (in our study, simulated failed predation). However, the mechanism inducing increased newt toxicity remains unclear. In most metazoans, TTX is produced by endosymbiotic bacteria rather than the host species itself. Because it remains undetermined how salamandrids produce TTX, the observed differences in TTX concentrations between laboratory- and stream- reared larvae and the rapid increases measured in adult *Taricha* could be due to TTX-producing endosymbionts responding to changes within the host environment. Alternatively, *Taricha* and related salamandrids may be uniquely capable of independently regulating chemical defenses in response to predator stimuli or physiological cues.

The results of our study align with broad theoretical models^31^ and the outcomes of numerous empirical studies on amphibian trade-offs that demonstrate increased defenses impart a cost^32–34^. Even though larvae were not reared in their native geographic stream, the initial similarity of TTX concentrations and body condition between siblings (Fig. 3, Supplemental Fig. 2), coupled with the significant temporal divergence of these traits, demonstrate that the phenotype is inducible. If such differences are not a result of endosymbionts, it suggests that larvae may have differentially allocated resources for chemical defenses. Just as theory predicts that plant defense morphology is constrained by a physiological trade-off between growth and differentiation^35^, chemically-defended animals appear to face similar limits, which for amphibians likely will be linked to delayed life history tradeoffs^36^. The costs associated with an induced defense can also limit a phenotype^37^ and as such, the negative relationship we observed between body condition (a loss of 20% BMI) and TTX concentrations of adult males (a 3–5 fold gain) implies that increased chemical defenses may be bounded by the toll such a response demands. Given the ubiquity of plastic phenotypes^38–41^ and chemically-defended amphibians, the induced response and associated cost is likely common^42, 43^. Because amphibian fitness is tightly linked to body condition^44, 45^ maintaining increased chemical defenses over sustained periods could have severe fitness consequences. If the cost is too steep, it may impede growth and feeding. Such scenarios may be most severe where chemically-defended amphibians coexist with recently introduced non-native predators^46^ and contribute to the decline of presumably well-defended amphibians^47^.

Our results fit within the theoretical prediction that inducible defenses will evolve when prey can reliably detect a predator or stimulus^48, 49^. The pattern of chemical defense increases, as well as our observations that only individuals we repeatedly sampled maintained elevated defenses, indicates that *T. torosa* reliably assess risk. In accordance with theory that predicts inducible traits will evolve when prey can survive predator attacks, predatory snakes will reject the most toxic newts that often survive these failed predation attempts^50^. If skin sampling is a reasonable proxy for predator attacks, then wild populations should similarly increase chemical defenses in response to attempted predation. We speculate that the observed initially low concentrations of TTX (Fig. 1d, hollow diamonds, grey squares; Fig. 1e grey squares; Fig. 2) are maintained with relatively little cost as a safeguard against initial attacks, which then can rapidly increase following a failed predation attempt.

Extensive geographic characterizations of amphibian chemical defense phenotypes^13, 14^ have assumed that such phenotypes are fixed on landscapes and respond to selection pressures on evolutionary time scales. However, our results indicate that such phenotypes are inducible. Although plasticity of chemical defenses in plants^51^ and invertebrates^52–54^ have played a large role in our understanding of the evolutionary and ecological basis of chemical defenses and secondary compounds, documenting plasticity of amphibian chemical defenses has remained relatively elusive. Our results demonstrate that environmental variation can immediately affect animal chemical defense levels with substantially large phenotypic and ecological consequences, especially to predator-prey relationships (Figs 2 and 3), and argue that the long-term evolutionary dynamics of amphibian chemical defenses are more complex than previously considered^14, 15, 55^. Ultimately, the responses of our study populations and the observed similarities across life history stages provide a strong empirical foundation to predict the rate, direction, magnitude, and costs of such phenotypic changes, and as such evince, at least in salamandrids, and likely many toxic animals, that chemical defenses are inducible.

## Methods

### Experimental populations and sites

Two experiments were performed using males from two breeding populations of adult newts (*Taricha torosa*) from near opposite ends of their distributional range (Fig. 1; *Supplementary Information*). Newts from central California (*n* = 30, ~10% of the breeding population; 37° 0′14.37″ N, 121°40′52.09″ W) were collected and transported to a study site in southern California as part of a 30-day *ex situ* experiment. This central California population has served as a study population in previous studies of newt chemical defenses^18^. Across the southern California range of *T. torosa*, the species is recognized as a *Species of Special Concern* due to precipitous population declines^56^. Therefore, these newts were not captured and removed from their breeding site. Instead, an *in situ* experiment was conducted using flow-through mesocosms installed throughout their breeding stream (*n* = 9; ~20% of the breeding population; 34° 3′25.54″ N, 118°38′12.46″ W). Geographically distant populations were selected to ensure that any discernable response was shared between populations and likely not due to local variation.

### Quantifying tetrodotoxin

To quantify TTX concentrations, we followed established protocols to collect 2 mm skin samples or larvae and quantitate TTX following^57^ (*Supplementary Information*). Estimates of whole newt dermal TTX concentrations (*whole newt toxicity*) were calculated using methods outlined in ref. 55 with initial and final repeatedly-sampled (group A) and *in situ* individual TTX concentrations and normalized s.e.m.^58^.

### *Ex situ* experiment

Males (n = 30) were randomly assigned to one of ten outdoor mesocosms (60 cm × 34 cm × 42 cm (*l* × *w* × *d*), 3 males per mesocosm). The ten mesocosms were divided into two sampling treatments (repeated sampling/group A and stratified sampling/group B) of 5 replicates (15 males per group) (Fig. 1b). Animals were measured (snout to vent length; SVL) and weighed (±0.1 mg) at the start of the experiment, and the first, second, or third toe trimmed from the front left foot^59^ to identify individuals within a mesocosm.

To test whether the chemical defense phenotype is rapidly responsive across short temporal scales and if environmental differences cause predictable changes to the direction and magnitude of the TTX phenotype, the groups were differentially sampled using a temporally stratified experimental design (Fig. 1b, *Supplementary Information*). Individuals in the repeated sampling treatment (group A, *n* = 15) were first sampled at the collection site on day 0, then every 6^th^ day over the 30-day period, thereby providing 6 samples per individual. Individuals in the stratified sampling treatment (group B, *n* = 15) were sampled in a temporally stratified pattern, such that a mesocosm of newts was sampled for the first time every 6^th^ day. Thus, the number of times individuals in this treatment were sampled depended upon mesocosm (Fig. 1b). For example, newts in mesocosm B1 were sampled initially on day six, then every 6^th^ day over the remaining 30-day period, whereas newts in mesocosm B5 were sampled for the first time and only time on the final day of the experiment. In both groups, newts were fed *ad libitum* and individuals were weighed each time they were sampled. Throughout the experiment, temperature and pH were logged in mesocosms.

### *In situ* experiments

Males (*n* = 9) were collected from breeding pools and placed in one of three flow-through mesocosms installed in the breeding stream (*Supplementary Information*). At the start of the experiment, each newt was weighed, SVL measured, and a skin sample taken from the dorsolateral posterior region. Newts were marked as detailed above, repeatedly sampled and weighed every 6^th^ day following methods described for repeatedly sampled newts (group A) in our *ex situ* experiment. The *in situ* experiment lasted 24 days and concluded at the end of the local breeding period.

### Wild non-captive newts

To evaluate the potential effect of captive conditions on the chemical defense phenotype, we sampled and marked (to prevent resampling) free-ranging wild newts from our sampling sites (central California (n = 13): days 12, 30; southern California (n = 20): days 0, 6, 12, 18, 24) and compared these concentrations to captive newts from the population (*Supplementary Information*). Wild and captive animals were sampled on the same calendar days to control for any possible temporal variation in TTX. We consider the TTX concentrations of these randomly sampled wild, free-ranging males from both study sites to be a representation of mean male TTX concentrations of the population at the time of sampling^21^.

### Experimental sampling of embryo and larval TTX concentrations

We collected egg masses (*n* = 15) from our central California site and reared siblings from the same egg mass in two different environments (stream or laboratory) to determine if larval newt TTX concentrations would differ as a result of the environment. Because siblings had similar TTX concentrations within egg masses (*n* = 5; *Supplementary Information*), we divided the remainder of egg masses (*n* = 10) into roughly equal halves containing on average 12 embryos. Siblings from one half of an egg mass were reared in a laboratory closed mesocosm while siblings from the corresponding half were reared in flow-through mesocosms installed in our study stream in southern California (*Supplementary Information*).

To determine if phenotypic responses occur in larval sibling chemical defenses, three larvae from each replicate in each environment were sampled at days 0, 21, and 42 post-hatch (*Supplementary Information*). Larvae were placed in 1 mL vials containing 300 μl of 0.1 M HOAc and stored at − 80 °C for five days until TTX was extracted. Before extracting TTX, larvae were weighed (± 0.01 mg) and digitally photographed for subsequent measurement of body length and developmental stage^60^ (*Supplementary Information*). Although larvae were not reared in their native stream, any difference in phenotypic responses between siblings nonetheless demonstrates that the phenotype is inducible, and would suggest that environmental variation, including any stress associated with captive versus natural conditions affects chemical defenses.

### Statistical analyses

#### *Ex situ* experiment

Because time and sampling are confounded, we did not use a single model to analyze whether experimental sampling and time in captivity affected TTX concentrations. Instead we used a multistep model to assess potential differences in chemical defenses between sampling treatments (repeated sampling/group A; stratified sampling/group B) in our *ex situ* experiment. We created four models in *R* ^61^ for use with the package *nlme* ^62^; *Supplementary Information*, Supplementary Fig. 1) that were implemented in a multi-step approach. All models treated log-transformed TTX concentrations as the response variable and coded *individual* as a random effect to account for repeated measures and to control for among-individual variation (*Supplementary Information*, Supplementary Fig. 1). The first model assessed broadly if any patterns in chemical defenses could be explained by water quality predictors (*temperature* and *pH)*, sampling treatment (*group* – repeated sampling (group A) and stratified sampling (group B)), body condition, or time in captivity (*days*). If predictors for body condition or water quality metrics were statistically significant (P < 0.05) and *group* was not (P > 0.05), we concluded that sampling had no effect on chemical defenses. If predictors for body condition or water quality were not significantly associated with TTX concentration, but sampling treatment or time were, we ran a second model to test whether chemical defenses in the two sampling treatments significantly differed from one another over time. For this comparison, we subset data by *group* to perform two independent analyses of TTX concentrations - one with the repeatedly-sampled newts in group A, the other with all newts in group B, and included a continuous predictor for time (*days*). A significant difference in one group but not the other suggested that sampling affected chemical defenses.

As a result of the sampling regime in the stratified sampling treatment (group B), we hypothesized that chemical defenses might differ between initially-sampled (B_i_) and repeatedly sampled (B_r_) newts. The third model evaluated this by sub-setting *group* (B_i_ and B_r_) and testing if TTX concentrations differed through time (*days*) in both data sets. A significant difference in TTX concentrations through time in repeatedly-sampled newts (B_r_) but not initially-sampled newts (B_i_) would suggest that sampling also affected chemical defenses in this treatment. The last model tested if captive conditions affected chemical defenses by statistically comparing TTX concentrations of initially-sampled (B_i_) newts to non-captive wild newts that were sampled on the same days in central California. This model also included a predictor to test for the effect of body condition. No random effects were included because no individuals in either group were repeatedly sampled.

To assess if chemical defenses and toxicity changed in repeatedly-sampled newts (group A), we used two separate paired two-tailed *t* tests in *R* to evaluate if initial and final individual TTX concentrations (nmol mg^−1^) and log-transformed estimated whole newt toxicity (mg TTX) statistically differed.

#### *In situ* experiment

We first determined if stream-captive newt chemical defenses changed over the duration of the experiment using a mixed-effect model in *R* with the package *nlme*. The model included a continuous predictor for time (*days*) and a random intercept for individual, with log-transformed TTX concentrations coded as the response variable. Next, we tested whether stream-captive chemical defenses differed from wild newt chemical defenses by updating the first model to include TTX values from days when both groups were sampled (*days* 6, 12, 18, 24) and the predictors for body condition and group (*location*: wild or stream-captive). As with *ex situ* analyses, we used paired t-tests to determine if initial and final stream captive newt chemical defenses and toxicity differed.

#### Larvae

To test whether larval chemical defenses differed between laboratory- and stream- reared siblings, we evaluated the effect of larval environment (*location*: laboratory or stream), time between hatching (*days;* 0, 21, 42), and whole larva body condition (*body condition*) on TTX concentrations by coding these predictors as a three-way interaction term using *R* in the package *nlme* (*Supplementary Information*). Egg mass was coded as a random effect to control for potential correlations within and variation between egg masses.

In southern California, *T. torosa* is a declining Species of Special Concern and population sizes are often small. Thus, we performed permutation tests on all models for each experiment to strengthen our confidence in our statistical analyses. The package *pgirmess* ^63^ was used in *R* to permute 1,000 Monte Carlo simulations of each individual model. Resultant test statistics were evaluated along with our model results.

### Data availability

The data and code required to replicate results will be archived in the Dryad Digital Repository.

### Ethics Statement

All protocols were approved by the Pepperdine IACUC committee and all ethical regulations followed throughout the entirety of each experimental process.

## Electronic supplementary material

Supplementary Information

## References

[1] Smith TB (1990). Resource use by bill morphs of an African finch: evidence for intraspecific competition. Ecology.

[2] Tilman D, Wedin D (1991). Plant traits and resource reduction for five grasses growing on a nitrogen gradient. Ecology.

[3] Bourke P, Magnan P, Rodríguez MA (1999). Phenotypic responses of lacustrine brook charr in relation to the intensity of interspecific competition. Evolutionary Ecology.

[4] Pigliucci, M. Phenotypic plasticity: beyond nature and nurture (syntheses in ecology and evolution). Johns Hopkins University Press, Baltimore, Maryland, USA (2001).

[5] Dudley SA, Schmitt J (1996). Testing the adaptive plasticity hypothesis: density-dependent selection on manipulated stem length in Impatiens capensis. American Naturalist.

[6] Hartle, D. L., & Clark, A. G. Principles of population genetics. Sunderland: Sirauer Associates (1997).

[7] Van Buskirk J, Relyea RA (1998). Natural selection for phenotypic plasticity: predator-induced morphological responses in tadpoles. Biological Journal of the Linnean Society.

[8] Kats LB, Dill LM (1998). The scent of death: chemosensory assessment of predation risk by prey animals. Ecoscience.

[9] Trussell GC, Smith LD (2000). Induced defenses in response to an invading crab predator: an explanation of historical and geographic phenotypic change. Proceedings of the National Academy of Sciences.

[10] Dahl J, Peckarsky BL (2002). Induced morphological defenses in the wild: predator effects on a mayfly, Drunella coloradensis. Ecology.

[11] Baldwin IT, Halitschke R, Paschold A, Von Dahl CC, Preston CA (2006). Volatile signaling in plant-plant interactions: “talking trees” in the genomics era. Science.

[12] Karban, R. & Baldwin, I. T. Induced responses to herbivory. University of Chicago Press, Chicago (1997).

[13] Brodie ED, Ridenhour BJ, Brodie ED (2002). The evolutionary response of predators to dangerous prey: hotspots and coldspots in the geographic mosaic of coevolution between garter snakes and newts. Evolution.

[14] Kraemer AC, Serb JM, Adams DC (2015). Model toxin level does not directly influence the evolution of mimicry in the salamander Plethodon cinereus. Evolutionary Ecology.

[15] Yotsu-Yamashita M, Mebs D, Kwet A, Schneider M (2007). Tetrodotoxin and its analogue 6-epitetrodotoxin in newts (Triturus spp.; Urodela, Salamandridae) from southern Germany. Toxicon.

[16] Yotsu-Yamashita M (2012). Variability of tetrodotoxin and of its analogues in the red-spotted newt, Notophthalmus viridescens (Amphibia: Urodela: Salamandridae). Toxicon.

[17] Geffeney SL, Fujimoto E, Brodie ED, Ruben PC (2005). Evolutionary diversification of TTX-resistant sodium channels in a predator–prey interaction. Nature.

[18] Hanifin CT, Brodie ED, Brodie ED (2008). Phenotypic mismatches reveal escape from arms-race coevolution. PLoS Biol.

[19] Hanifin CT, Brodie ED (2002). Tetrodotoxin levels of the rough-skin newt, Taricha granulosa, increase in long-term captivity. Toxicon.

[20] Cardall BL, Brodie ED, Hanifin CT (2004). Secretion and regeneration of tetrodotoxin in the rough-skin newt (Taricha granulosa). Toxicon.

[21] Bucciarelli GM, Green DB, Shaffer HB, Kats LB (2016). Individual fluctuations in toxin levels affect breeding site fidelity in a chemically defended amphibian. Proc. R. Soc. B.

[22] Watson RT, Mathis A, Thompson R (2004). Influence of physical stress, distress cues, and predator kairomones on the foraging behavior of Ozark zigzag salamanders, Plethodon angusticlavius. Behavioural Processes.

[23] Davis AK, Maerz JC (2008). Comparison of hematological stress indicators in recently captured and captive paedomorphic mole salamanders, Ambystoma talpoideum. Copeia.

[24] Johansson F, Lederer B, Lind MI (2010). Trait performance correlations across life stages under environmental stress conditions in the common frog, Rana temporaria. PLoS One.

[25] Turner AM, Montgomery SL (2003). Spatial and temporal scales of predator avoidance: experiments with fish and snails. Ecology.

[26] Wolfe GV, Steinke M (1996). Grazing‐activated production of dimethyl sulfide (DMS) by two clones of Emiliania huxleyi. Limnology and Oceanography.

[27] Matz C, Kjelleberg S (2005). Off the hook–how bacteria survive protozoan grazing. Trends in microbiology.

[28] Mithöfer A, Boland W (2012). Plant defense against herbivores: chemical aspects. Annual review of plant biology.

[29] Bollens SM, Frost BW, Thoreson DS, Watts SJ (1992). Diel vertical migration in zooplankton: field evidence in support of the predator avoidance hypothesis. Hydrobiologia.

[30] Brönmark C, Miner JG (1992). Predator-induced phenotypical change in body morphology in crucian carp. Science.

[31] Clark CW, Harvell CD (1992). Inducible defenses and the allocation of resources: a minimal model. American Naturalist.

[32] Newman RA (1992). Adaptive plasticity in amphibian metamorphosis. BioScience.

[33] Van Buskirk J (2000). The costs of an inducible defense in anuran larvae. Ecology.

[34] Miner BG, Sultan SE, Morgan SG, Padilla DK, Relyea RA (2005). Ecological consequences of phenotypic plasticity. Trends in Ecology & Evolution.

[35] Herms DA, Mattson WJ (1992). The dilemma of plants: to grow or defend. Quarterly review of biology.

[36] Searcy CA, Gray LN, Trenham PC, Shaffer HB (2014). Delayed life history effects, multilevel selection, and evolutionary trade‐offs in the California tiger salamander. Ecology.

[37] Tollrian, R. and D. Harvell. editors 1999. The ecology and evolution of inducible defenses. Princeton University Press, Princeton, New Jersey, USA.

[38] Schlichting, C. D. & Pigliucci, M. Phenotypic evolution: a reaction norm perspective. Sinauer Associates Incorporated (1998).

[39] Relyea RA (2002). Competitor-induced plasticity in tadpoles: consequences, cues, and connections to predator-induced plasticity. Ecological Monographs.

[40] Hayes RA, Crossland MR, Hagman M, Capon RJ, Shine R (2009). Ontogenetic variation in the chemical defenses of cane toads (Bufo marinus): toxin profiles and effects on predators. Journal of chemical ecology.

[41] Kishida O, Trussell GC, Nishimura K (2007). Geographic variation in a predator-induced defense and its genetic basis. Ecology.

[42] Benard MF, Fordyce JA (2003). Are induced defenses costly? Consequences of predator-induced defenses in western toads, Bufo boreas. Ecology.

[43] Saporito RA, Donnelly MA, Garraffo HM, Spande TF, Daly JW (2006). Geographic and seasonal variation in alkaloid-based chemical defenses of Dendrobates pumilio from Bocas del Toro, Panama. Journal of chemical ecology.

[44] Semlitsch RD, Scott DE, Pechmann JH (1988). Time and size at metamorphosis related to adult fitness in Ambystoma talpoideum. Ecology.

[45] McCollum, S. A., & Van Buskirk, J. Costs and benefits of a predator-induced polyphenism in the gray treefrog Hyla chrysoscelis. *Evolution*, 583–593 (1996). 10.1111/j.1558-5646.1996.tb03870.x 28568914

[46] Gamradt SC, Kats LB (1996). Effect of introduced crayfish and mosquitofish on California newts. Conservation Biology.

[47] Preston DL, Henderson JS, Johnson PT (2012). Community ecology of invasions: direct and indirect effects of multiple invasive species on aquatic communities. Ecology.

[48] Dodson S (1989). Predator-induced reaction norms. Bioscience.

[49] Thompson JD (1991). Phenotypic plasticity as a component of evolutionary change. Trends in Ecology & Evolution.

[50] Williams BL, Brodie ED, Brodie ED (2003). Coevolution of deadly toxins and predator resistance: Self-assessment of resistance by garter snakes leads to behavioral rejection of toxic newt prey. Herpetologica.

[51] Karban R, Myers JH (1989). Induced plant responses to herbivory. Annual Review of Ecology and Systematics.

[52] Moranz R, Brower LP (1998). Geographic and temporal variation of cardenolide-based chemical defenses of queen butterfly (Danaus gilippus) in northern Florida. Journal of Chemical Ecology.

[53] Fordyce JA, Marion ZH, Shapiro AM (2005). Phenological variation in chemical defense of the pipevine swallowtail, Battus philenor. Journal of chemical ecology.

[54] Wood SA (2012). Tetrodotoxin concentrations in Pleurobranchaea maculata: temporal, spatial and individual variability from New Zealand populations. Marine drugs.

[55] Hanifin CT, Brodie ED (2004). A predictive model to estimate total skin tetrodotoxin in the newt Taricha granulosa. Toxicon.

[56] Thomson, R. C., Wright A. N.,and Shaffer H. B. California Amphibian and Reptile Species of Special Concern. Univ of California Press (2016).

[57] Bucciarelli GM, Li A, Kats LB, Green DB (2014). Quantifying tetrodotoxin levels in the California newt using a non-destructive sampling method. Toxicon.

[58] Morey RD (2008). Confidence intervals from normalized data: A correction to Cousineau (2005). Reason.

[59] Funk WC, Donnelly MA, Lips KR (2005). Alternative views of amphibian toe-clipping. Nature.

[60] Harrison, R. G. Harrison stages and description of the normal development of the spotted salamander, Ambystoma punctatum (Linn.). *Organization and Development of the Embryo*, 44–66 (1969).

[61] R Development Core Team. 2016. R: A language and environment for statistical computing. Vienna, Austria: R Foundation for Statistical Computing.

[62] Pinheiro J, Bates D, DebRoy S, Sarkar D, Core Team R (2015). *nlme: Linear and Nonlinear Mixed Effects Models*. R package version.

[63] Giraudoux P (2012). pgirmess: Data analysis in ecology. R package version.

[64] Wickham H (2009). ggplot2: elegant graphics for data analysis.

