# Supplementary material for: An amphibian chemical defense phenotype is inducible across life history stages
Source: Sci Rep. 2017 Aug 15;7:8185. doi: 10.1038/s41598-017-08154-z (PMC5558003; doi:10.1038/s41598-017-08154-z)
Supplement: Supplementary file 1 — Supplementary Information [file 41598_2017_8154_MOESM1_ESM.pdf]

Supplementary Information

An amphibian chemical defense phenotype is inducible across life history stages

Gary M. Bucciarelli, H. Bradley Shaffer, David B. Green, and Lee B. Kats

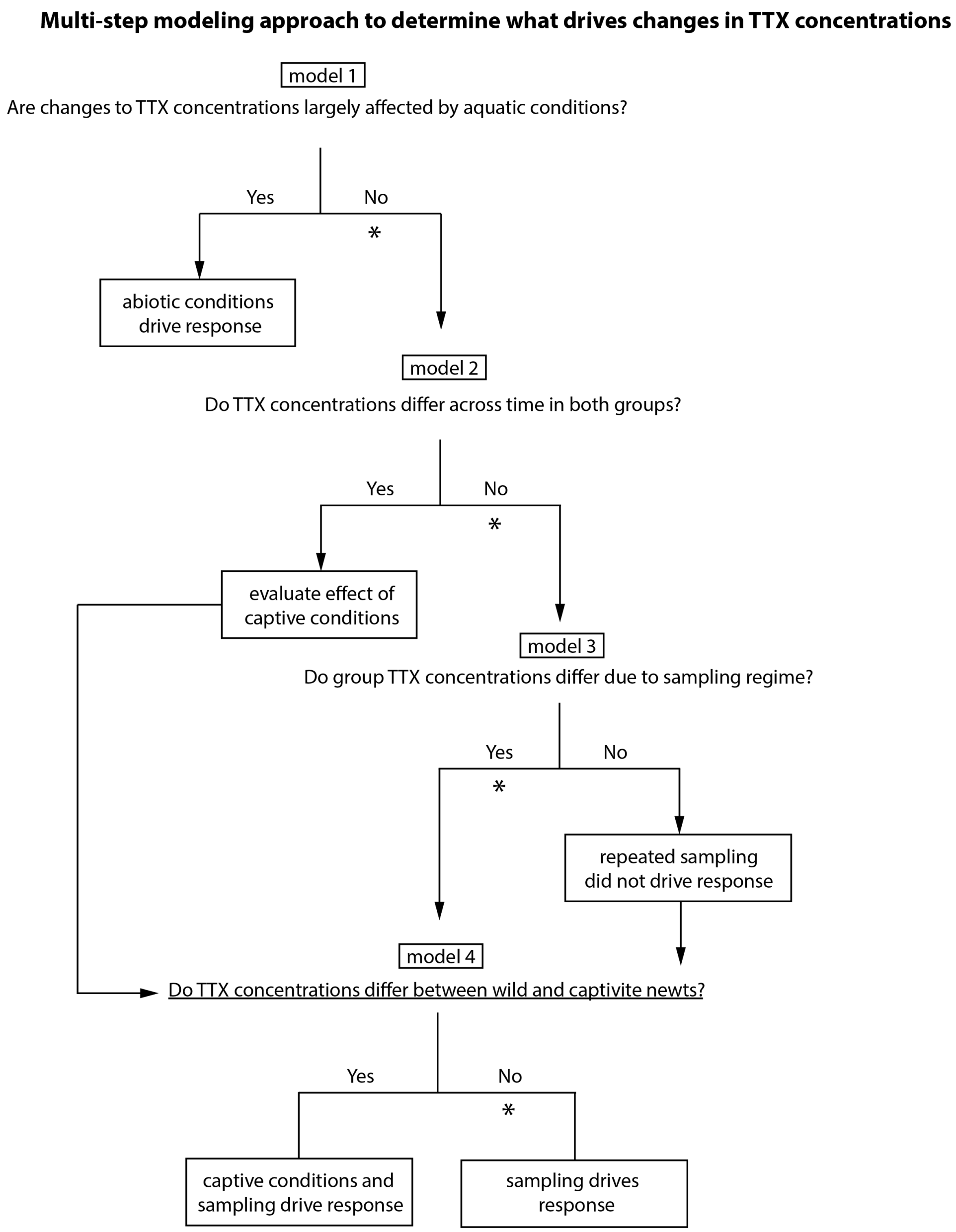

Supplementary Fig. 1. The multistep modeling approach to assess the effects of sampling and captive conditions on newt chemical defenses utilized four mixed-effects models. The flow chart is marked (*) to show the overall results and interpretation from each model. Overall, changes in TTX concentrations in newts from central California appear to be driven by repeated sampling and not captivity.

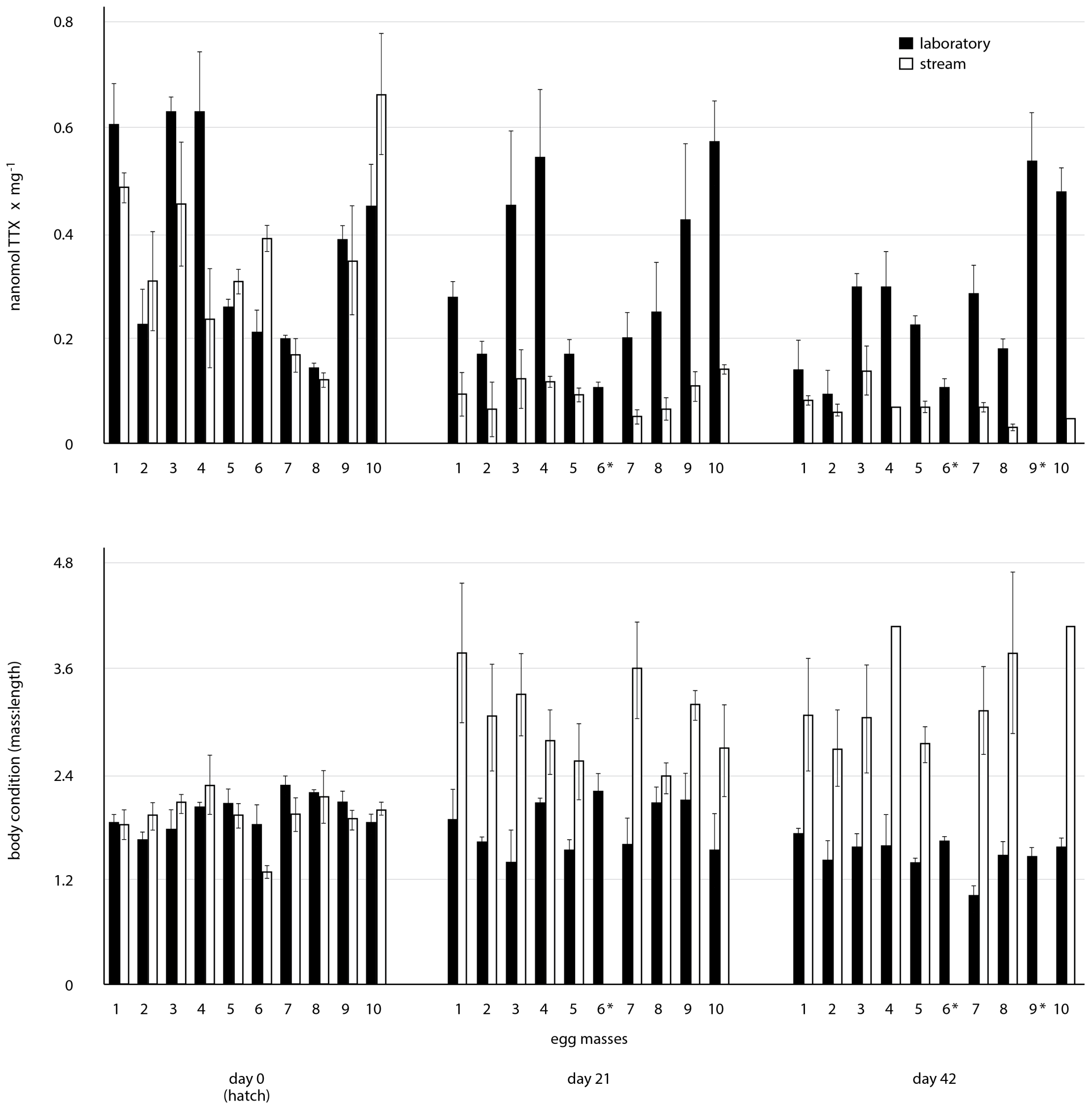

Supplementary Fig. 2. Mean TTX concentrations and body condition (± s.e.m) of siblings from ten egg masses over a 42-day period. Each numbered pair shows the larval TTX concentrations or body condition value of siblings from the same egg mass over the 42-day period. Larvae were lost from stream egg mass 6 prior to day 21 and all individuals were sampled from stream egg mass 9 by day 42 (*). Values without s.e.m. bars represent the only remaining larva.

Supplementary Methods

Collection and transportation of newts – Adult males from central California were collected and individually placed in polyethylene bags filled with ~ 500 ml of stream water maintained at 9 °C (± 1 °C) and transported to our study site in southern California Malibu, CA. Adults were considered to be any individuals with snout vent length that exceeded 60 mm. The sex of each male was determined using sexually dimorphic morphological characteristics that are easily distinguishable when adults are in breeding condition, which includes taller tail heights, increased limb sizes, and smooth versus granular skin.

To estimate the number of individuals per population required to detect a pre- and post-sampling effect, *a priori* power analyses were performed using the G*Power application (Faul et al. 2007). Once at population collection sites, these estimates (n = 17 per group) exceeded the maximum number of individuals we could collect for our experiments based on permit guidelines from the California Fish and Wildlife. Therefore, we collected the maximum number of individuals in our central California (n = 30; ~ 20% of the breeding adults) and southern California (n = 9; ~10% of the breeding adults) populations.

TTX extraction and quantitation – In summary (Bucciarelli et al. 2014), tissue, embryos, or larvae were weighed then macerated. Solutions were boiled for 5 minutes, cooled for 5 minutes, then microcentrifuge filtered for 20 minutes at 13,000 rpm. Filters were filled with 100 µl of 0.1 M AcOH and centrifuged for another cycle of 20 minutes. The

supernatant was collected, brought to a final volume of 1 mL, and stored at -80 °C until the sample was analyzed via high performance liquid chromatography with fluorescence detection (HPLC-FLD). Peak values from chromatographs were used to derive concentrations of nanomoles TTX per milligram of material based on a standard curve.

Ex situ *experiment* – Mesocosms were each filled with ~ 70 L of filtered well water at 10 °C. Mesocosms were located on the north side of a 3.5 m high shade structure to minimize direct exposure to sunlight and reduce variation in water temperature. A concrete block (41 cm × 15 cm × 20 cm, $l \times w \times h$) was placed in the center of each mesocosm as a terrestrial resting site. Every six days, one third of the water was replaced in each mesocosm. All water quality parameters were logged using Horiba monitors and probes (U5100, Kyoto, Japan).

Newts were initially sampled on the left posterior dorsolateral area and additional skin samples were collected from alternating dorsolateral areas. When an individual had been sampled once on each side, subsequent sampling was performed anteriorly with each skin sample centered 1.5 cm from the previous sample.

In situ *experiment* – Mesocosms were fabricated from acrylic with 2 mm mesh on all four sides and installed in the stream at 75 m intervals and a depth of ~ 35 cm to allow water to pass through. Once in place, waterborne fluorescein was injected upstream of mesocosms to confirm that water flowed through each enclosure, which ensured that environmental conditions inside and outside of mesocosms were roughly equivalent.

Animals were marked as previously described by clipping one forelimb digit and

provided the same concrete structure and quantities of food as central California newts in the *ex situ* experiment. In addition, we added ~ 400 g of substrate in the form of stream cobble and leaf litter to each stream mesocosm. Although we manually removed any predatory macroinvertebrates from this material, it is likely that additional prey were available to adults in this leaf litter.

Non-captive newts – On days 12 and 30 of our *ex situ* experiment, we returned to the site of our central California population to sample and weigh random wild, free-ranging males from this breeding population. In our southern California population, we sampled and weighed randomly selected non-captive males each day that we sampled captive newts in the *in situ* experiment. In both experiments, wild, non-captive newts were never repeatedly sampled. This was ensured by collecting tail tips (for subsequent DNA analyses) as a form of marking. Ultimately, if captive conditions had little effect on TTX concentrations, there should be no significant difference between wild, non-captive and captive newt TTX concentrations.

Analysis of embryo TTX within egg masses – Female *T. torosa* oviposit egg masses that typically contain 20 - 30 eggs (Brame 1968). We collected egg masses at the onset of ovipositing from Central California and transported them in stream water at 9 °C to the laboratory. Egg masses not immediately used to assess embryo TTX concentrations were individually placed in 10 × 10 cm acrylic bins filled with 5-μm-filtered, dechlorinated tap water at a temperature of 9 °C and housed for 24 hours in a walk-in refrigerator on a 12:12 h light:dark cycle.

To determine whether potential differences of TTX concentrations between larvae reared in laboratory or stream environments may be due to inherent variation within an egg mass, we quantified the TTX in embryos from egg masses (n = 5) and statistically tested for significant differences of TTX between embryos within an egg mass. We dissected all embryos from each egg mass and embryos were rinsed, weighed, placed in 300 μL of 0.1 M HOAc, and stored at -80 °C until TTX was extracted and quantitated using high performance liquid chromatography with fluorescence detection (Bucciarelli et al. 2014). In *R*, the derived TTX concentrations for each egg mass were randomly divided into two groups and values statistically compared using a t-test. The process was permuted 1,000 times for each of the five egg masses and the resulting mean p-value recorded. We found strong support for no significant difference of TTX concentrations within any of the egg masses, with p-values ranging from 0.68 – 0.99.

Experimental sampling of larvae – One half of each divided egg mass was placed in a semi-transparent acrylic mesocosm (37 × 21 × 16 cm, l × w × d) filled with 4 L of 5-μm-filtered, dechlorinated tap water that was maintained in a laboratory walk-in cold room. The corresponding half was housed in a stream mesocosm at our study stream in southern California. The 10 mesocosms were installed at roughly 25 m intervals throughout a 300 m stretch of the creek. These stream mesocosms were structurally similar to laboratory mesocosms, but had 2 mm mesh sides to create a flow-through system. In each stream mesocosm, we added stones and fresh leaf litter for larvae to use as cover. However, we provided no additional materials in the laboratory mesocosms. Larvae in both environments were fed *ad libitum* brine shrimp and blackworms (*Lumbriculus*

variegatus). To ensure similar hatch times, temperatures in laboratory mesocosms were consistently maintained to match measured stream mesocosm temperatures. In both environments, all larvae hatched within a 48-hour window. Our experimental sampling period began at time zero, which we recognize as the day when all larvae across both environments had hatched. On average, egg mass halves contained 12 embryos on average (range: 12 – 15). This sampling scheme would have provided up to 4 time points for sampling, but an average of 2.4 larvae died across each treatment during the experimental period.

Larval measurements, morphology, and developmental stages – Larvae were weighed (± 0.01 mg), transferred to a translucent acrylic plate, and photographed with a mounted digital camera (Nikon, D90). All images were captured in JPEG format, processed in *ImageJ* software (*version 1.47*), and calibrated in *ImageJ* to 1 mm using a 5-cm measuring tape affixed to the work surface in each image. Larvae were digitally measured from head to tail-tip and morphological features inspected to determine developmental stage.

Statistical analyses

For all analyses, we chose to use mixed-effects models fit with linear regression in *nlme* so that temporal predictors could be treated as continuous, and because such models can withstand unbalanced data, clustered data and repeated measures in the same model, as well as variation in the number of measured observations for each individual (Verbeke &

Molenberghs 2009). Given the observed break points in our data, we considered using piecewise regressions. However, ΔAIC values of piecewise and linear models differed by values less than 1. Therefore, we proceeded with our linear models. All data for the response variable were derived from peak area values from HPLC analyses and log-transformed to normalize the residuals. To select the appropriate random effects, we generated a null model with the two potential random effects of *individual* and *mesocosm* nested accordingly within the model. An assessment of variation of TTX concentrations across mesocosms in the *ex situ* experiment showed that the standard deviation of *mesocosm* was always much less than across *individual*. As a result, we used only *individual* as a random effect in the models.

Larval development and TTX concentrations – To ensure that differences in larval TTX concentrations were not a result of differences in development, we performed a series of t - tests to determine if developmental stages differed between laboratory and stream larvae on any of our sampling days. Larvae at 21-days post-hatch were beyond the final Harrison larval stage (detectable front limb buds). Therefore, we created numerical values to denote whether larvae had developed hind limb buds, fully developed forelimbs, fore limbs with digits, fully developed hind limbs, or hind limbs with digits. These data were used to test for potential developmental differences between the two groups by comparing development values at each time point (0, 21, and 42 days). If development differed between laboratory and stream larvae on a specific day of sampling, then t-tests were used to determine if their TTX concentrations also significantly differed.

Tests of larval developmental stages and TTX concentrations at the time of hatching did not significantly differ (*developmental stage*: $p = 0.56$, $t = 0.58$, $df = 58$; *TTX concentrations*: $p = 0.60$, $t = 0.52$, $df = 58$, *stream* $\bar{x} = 0.35 \pm 0.03$, *laboratory* $\bar{x} = 0.37 \pm 0.03$). However, developmental stages significantly differed between larval groups on 21-days ($p = 0.047$) and 42-days ($p = 0.006$). Therefore, we tested whether developmental stages at 21 days post-hatch and TTX concentrations of stream-reared larvae differed from TTX concentrations of laboratory-reared larvae at 42-days post-hatch. Developmental stages did not differ ($p = 0.42$), but their TTX concentrations did ($p < 0.001$; *stream* $\bar{x} = 0.10$ nmol/mg; *laboratory* $\bar{x} = 0.26$ nmol/mg). As a result, the difference in TTX concentrations between laboratory- and stream- reared larvae at the end of our experiment does not seem to be an effect of development.

Literature Cited

Brame, A. H. (1968). The number of egg masses and eggs laid by the California newt, Taricha torosa. *Journal of Herpetology*, **2**, 169-170.

Bucciarelli, G. M., Li, A., Kats, L. B., & Green, D. B. (2014). Quantifying tetrodotoxin levels in the California newt using a non-destructive sampling method. *Toxicon*, **80**, 87-93.

Faul, F., Erdfelder, E., Lang, A.G., & Buchner, A. (2007). G*Power 3: A flexible statistical power analysis program for the social, behavioral, and biomedical sciences. *Behavior Research Methods*, **39**, 175-191.

Verbeke, G., & Molenberghs, G. (2009). Linear mixed models for longitudinal data. Springer Science & Business Media.
